# Interleukin 33/ST2 Axis Components Are Associated to Desmoplasia, a Metastasis-Related Factor in Colorectal Cancer

**DOI:** 10.3389/fimmu.2019.01394

**Published:** 2019-06-21

**Authors:** Glauben Landskron, Marjorie De la Fuente López, Karen Dubois-Camacho, David Díaz-Jiménez, Octavio Orellana-Serradell, Diego Romero, Santiago A. Sepúlveda, Christian Salazar, Daniela Parada-Venegas, Rodrigo Quera, Daniela Simian, María-Julieta González, Francisco López-Köstner, Udo Kronberg, Mario Abedrapo, Iván Gallegos, Héctor R. Contreras, Cristina Peña, Guillermo Díaz-Araya, Juan Carlos Roa, Marcela A. Hermoso

**Affiliations:** ^1^Immunology Program, Innate Immunity Laboratory, Faculty of Medicine, Biomedical Sciences Institute, Universidad de Chile, Santiago, Chile; ^2^Research Sub-direction, Academic Direction, Clinica Las Condes, Santiago, Chile; ^3^Pathology Department, Faculty of Medicine, Pontificia Universidad Catolica de Chile, Santiago, Chile; ^4^Inflammatory Bowel Disease Program, Gastroenterology Department, Clinica Las Condes, Santiago, Chile; ^5^Cell and Molecular Biology Program, Faculty of Medicine, Institute of Biomedical Sciences, Universidad de Chile, Santiago, Chile; ^6^Coloproctology Department, Clinica Las Condes, Santiago, Chile; ^7^Coloproctology Surgery Department, Hospital Clinico Universidad de Chile, Santiago, Chile; ^8^Pathology Department, Hospital Clinico Universidad de Chile, Santiago, Chile; ^9^Department of Basic and Clinic Oncology, Faculty of Medicine, Universidad de Chile, Santiago, Chile; ^10^Medical Oncology Department, Ramon y Cajal University Hospital, IRYCIS, CIBERONC, Madrid, Spain; ^11^Molecular Pharmacology Laboratory, Faculty of Chemical Pharmaceutical Sciences, Universidad de Chile, Santiago, Chile

**Keywords:** colorectal cancer, cancer associated fibroblasts, interleukin 33, desmoplasia, epithelial-mesenchymal transition

## Abstract

In colorectal cancer (CRC), cancer-associated fibroblasts (CAFs) are the most abundant component from the tumor microenvironment (TM). CAFs facilitate tumor progression by inducing angiogenesis, immune suppression and invasion, thus altering the organization/composition of the extracellular matrix (i.e., desmoplasia) and/or activating epithelial-mesenchymal transition (EMT). Soluble factors from the TM can also contribute to cell invasion through secretion of cytokines and recently, IL-33/ST2 pathway has gained huge interest as a protumor alarmin, promoting progression to metastasis by inducing changes in TM. Hence, we analyzed IL-33 and ST2 content in tumor and healthy tissue lysates and plasma from CRC patients. Tissue localization and distribution of these molecules was evaluated by immunohistochemistry (using localization reference markers α-smooth muscle actin or α-SMA and E-cadherin), and clinical/histopathological information was obtained from CRC patients. *In vitro* experiments were conducted in primary cultures of CAFs and normal fibroblasts (NFs) isolated from tumor and healthy tissue taken from CRC patients. Additionally, migration and proliferation analysis were performed in HT29 and HCT116 cell lines. It was found that IL-33 content increases in left-sided CRC patients with lymphatic metastasis, with localization in tumor epithelia associated with abundant desmoplasia. Although ST2 content showed similarities between tumor and healthy tissue, a decreased immunoreactivity was observed in left-sided tumor stroma, associated to metastasis related factors (advanced stages, abundant desmoplasia, and presence of tumor budding). A principal component analysis (including stromal and epithelial IL-33/ST2 and α-SMA immunoreactivity with extent of desmoplasia) allowed us to distinguish clusters of low, intermediate and abundant desmoplasia, with potential to develop a diagnostic signature with benefits for further therapeutic targets. IL-33 transcript levels from CAFs directly correlated with CRC cell line migration induced by CAFs conditioned media, with rhIL-33 inducing a mesenchymal phenotype in HT29 cells. These results indicate a role of IL-33/ST2 in tumor microenvironment, specifically in the interaction between CAFs and epithelial tumor cells, thus contributing to invasion and metastasis in left-sided CRC, most likely by activating desmoplasia.

## Introduction

Colorectal cancer (CRC) is one of the most frequent types of cancer, with the third highest incidence in men and the second in women worldwide; with more than half of all cases occurring in developed countries (1). In Chile, crude death rate has duplicated in the past years, being the fourth most deadly cancer in men and third on women (1, 2). CRC is also a multifactorial pathology that occurs with the formation of a focus of aberrant crypt, progresses with the appearance of polyps, adenoma and finally carcinoma, which comprises the final stage of malignant epithelial transformation (3, 4).

The tumor microenvironment is a very complex structure represented by several cells, including tumor cells, resident fibroblasts, endothelial cells, and recruited macrophages/lymphocytes, that establish communications with each other and with tumor cells by means of soluble factors and cell-cell contact (5, 6). Depending on the context and the predominant cytokine profile, immune response can favor, or delay tumor progression (7, 8), and in some tumors a fibrotic response is also detected. Among all cell types, cancer associated fibroblasts (CAFs) are the most abundant stromal cells and can mediate a fibrotic response to a chronic inflammatory milieu (9).

During the tumor formation, not only the epithelial cells undergo changes, but also the stroma, with morphological alterations such as desmoplasia, angiogenesis and inflammatory or immune cell infiltration (10, 11). Desmoplasia corresponds to a stromal reaction to the tumor, where CAFs supply matrix remodeling molecules [e.g., tenascin, metalloproteinases (MMPs)] affecting extracellular matrix component deposition in the invasion front (12), and organizing protein fibers toward an ordered pattern favoring tumor cell migration (13).

In CRC, desmoplasia, together with advanced invasion stages and lymph node (LN) metastasis, constitutes an independent factor of poor prognosis (low free survival of recurrence at 5 years) (14, 15). In addition, desmoplastic reaction can be associated with tumor dedifferentiation, thus contributing to tumor invasion (16). A high α-smooth muscle actin (α-SMA) content reflects desmoplasia and is associated with poor prognosis (17), and also with high M2-type macrophage content (18), confirming the interaction previously described between CAFs and M2 macrophages in processes of fibrosis and tumor progression (19, 20).

IL-33 is a cytokine belonging to the family of IL-1, which is expressed not only in non-hematopoietic cells (fibroblasts, adipocytes, endothelial, smooth muscle, and epithelial cells), but also in macrophages and dendritic cells (21–23). The IL-33 receptor is called ST2 (encoded by *IL1RL1* gene), of which there are two variants: one membrane-anchored called ST2L or IL-33R, which exerts the cellular effects of IL-33 and a soluble variant, the sST2, lacks the transmembrane portion acting as decoy receptor of IL-33 (24). When IL-33 binds to the receptor complex formed by ST2L and the IL-1 receptor accessory protein (IL1RAcP), activation of a signaling pathway mediated by MAP kinases (mitogen-activated protein) and NF-κB takes place (21). In cells of the immune system (mast cells, nuocytes, or innate lymphoid cell type 2 and eosinophils), expressing ST2, IL-33 induces the synthesis and secretion of Th2-related cytokines (IL- 4, IL-13, or IL-5) (21, 25, 26).

Previously evaluated for its role as a pro-inflammatory cytokine in the pathophysiology of inflammatory bowel diseases, mainly ulcerative colitis (27), IL-33 has been implicated in tumorigenic processes in murine and *in vitro* models (28–30). Furthermore, elevated serum IL-33 levels have been detected in patients with lung, gastric, and hepatocellular cancer (30), although conversely, IL-33 activates CD8+ T lymphocytes and NK effector cells in the antitumor response in murine models of immunotherapy and lung and melanoma cancer, exerting a protective role (31, 32).

Moreover, IL-33 secreted by CAFs from patients with head and neck cancer has been associated with invasion by activating Epithelial to Mesenchymal Transition (EMT) (33), which emphasize the importance of studying IL-33 in metastasis.

In CRC, the IL-33/ST2 axis activates the tumor stroma fibroblasts promoting polyp formation in Adenomatous Poliposis Coli (APC)^Min/+^ mice model (34). In addition, IL-33, and total ST2 mRNA content were found elevated in tumor tissue of patients (adenomas > carcinomas) vs. normal tissue (35, 36), and were related to increased invasion and metastasis in CRC tumor cells and xenograft murine models of a IL-33-overexpressing tumor cell line (36). Additionally, high IL-33 immunoreactivity in metastatic CRC tumor cells has been associated with shorter survival (37), confirming sST2 as a protective tumorigenesis factor by counteracting protumoral IL-33 effects such as angiogenesis induction and modification of tumor microenvironment (38).

However, the content of sST2 is diminished in tumor tissue of CRC patients and inversely correlates with more advanced tumors, as well as IL-33 content correlation to tumor progression (38, 39), suggesting that IL-33/ST2 axis participates in the progression to CRC metastasis.

Regarding the role of IL-33/ST2 in CRC, studies showed activation of stroma in intestinal human myofibroblast cell lines and murine models of CRC stimulated both by IL-33 (34), however, the impact of IL-33/ST2 axis in desmoplasia of CRC patients is unknown. From this perspective, using a cohort of Chilean patients, we analyzed the association of IL-33/ST2 content and distribution with CRC progression and clinical/histopathological features (TNM staging, desmoplasia, tumor localization, among others) in tumor and healthy tissue.

## Patients and methods

### Patients

Samples from 62 patients (mean age 65.1 ± 14 years old, 38% women) with CRC were included from three health centers (Tissue Biobank of Universidad de Chile Clinical Hospital, Coloproctology Departments from Universidad de Chile Clinical Hospital and Clinica Las Condes) between 2015 and 2017. Patients undergoing surgery for tumor resection had to be older than 18 years old and not have received chemotherapy or neoadjuvant therapy prior to total or partial colectomy. Tumor staging was classified according to the TNM classification (The Union for International Cancer Control; UICC) (40).

Immediately after surgery, samples of fresh tumor including core and invasive front and healthy intestinal mucosa (at least 10 cm away from tumor) were macroscopically selected by a pathologist from each center in consensus to ensure homogeneity between samples. A small fragment from each selected tissue was stored at −80°C until posterior delivery to the Innate Immunity Lab for protein extraction. Biopsy size-samples of tumor and healthy tissue were fixated in 2% paraformaldehyde and embedded in paraffin for a tissue microarray (TMA) construction and immunohistochemistry analysis. Histology sections from primary tumor and healthy intestinal mucosa, metastatic and healthy LNs were also used for immunofluorescence analysis. Plasma samples from 34 CRC patients and 15 age-matched healthy controls were also obtained after signed informed consent. Clinical data from CRC patients and controls were also obtained for association analysis. Histological evaluation was conducted by a pathologist blinded to the patients' information.

The clinical and demographic information of CRC patients and controls is summarized in Table 1 and classified by the type of sample. Tumor localization proximal to the splenic flexure was defined was right-sided cancers and those at or distal to the splenic flexure as left-sided cancer (41). We were not able to obtain the same variety of samples from CRC patients; hence, different sample sizes are shown.

**Table 1:** Demographic characteristics from colorectal cancer patients included in study.

	**Total CRC patients**	**Frozen tissue**	**Plasma from CRC patients**	**Tissue microarray (TMA)**	**Plasma from healthy controls**
Sample size	**62**	41	34	22	**15**
Gender (F/M)	**24/38**	19/22	14/20	8/14	**7/8**
Age (range)	**65.1 (32–91)**	66.6 (32–91)	63.8 (42–89)	65.1 (33–85)	**50.7 (29–79)**
**Localization**					
Left-colon	**41**	26	24	14	
Right-colon	**21**	14	9	8	
**Tnm**					
1	**8**	5	4	5	
2	**22**	15	15	7	
3	**30**	20	15	8	
4	**1**	0	0	1	

*Bold values indicate the total number or age (range) of patients or controls*.

### Ethics Statement

This study was carried out in accordance with the recommendations of the Review Boards and Local Ethical Committees from Universidad de Chile Clinical Hospital, Tissue Biobank of Universidad de Chile and Clinica Las Condes, with written informed consent from all subjects. All subjects gave written informed consent in accordance with the Declaration of Helsinki and were identified only by codification established by their respective Center to keep anonymity. The protocol was approved by the Local Ethical Committees from Universidad de Chile Clinical Hospital, Tissue Biobank of Universidad de Chile and Clinica Las Condes.

### Cell Lines and Reagents

HT29 and HCT116 adenocarcinoma cell lines were kindly donated from Dr. Julio Tapia (Universidad de Chile, Santiago, Chile) and Dr. Jorge Toledo (Universidad de Concepcion, Concepcion, Chile), respectively. These cells were cultured in DMEM high-glucose cell culture media (Corning Life Sciences, Tewksbury, MA, USA) with 10% Fetal Bovine Serum (FBS, Gibco, Thermo Scientific, Waltham, MA, USA) + 100 U/ml penicillin, 100 μg/ml streptomycin (Gibco, Thermo Scientific, Waltham, MA, USA). Each cell passage was made with 0.025% trypsin/EDTA (Gibco, Thermo Scientific, Waltham, MA, USA) and sterile PBS (Sigma–Aldrich, St. Louis, MO, USA).

### Quantification of IL-33 and ST2 Protein From Tissue and Plasma

Frozen tumor and healthy adjacent tissues from patients with CRC were lysed with radioimmunoprecipitation (RIPA) buffer (Sigma–Aldrich, St. Louis, MO, USA) containing protease inhibitors (Roche Diagnostics GmbH, Mannheim, Germany) and lysates total protein was quantified with Pierce BCA Protein Assay Kit (Pierce Biotechnologies, Rockford, IL, USA). Plasma of CRC patients and healthy controls was collected from whole blood in BD Vacutainer® EDTA tubes. The determination of IL-33 and ST2 in tissue extracts and plasma were performed in duplicate by enzyme-linked immunosorbent assay (ELISA) DuoSet kits (R&D Systems, Minneapolis, MN, USA). The IL-33 and ST2 protein absorbance were read at 450 nm, by spectrophotometric analysis (Synergy 2, Biotek Instruments, Inc., Winooski, VT, USA). Protein levels from tissue extracts are expressed in pg/mg and normalized to the total protein content (mg), plasma results are expressed in pg/mL.

### Tissue Microarray (TMA) Assembly and Histological Characterization

A TMA was generated from formalin-fixed paraffin-embedded tissue from 31 patients with CRC. A representative zone was selected by a pathologist through careful observation of the Hematoxylin/Eosin stain. With a 2 mm diameter punch (Beecher Instruments, Silver Spring, MD, USA), cores from paraffin-embedded tissue were transferred to a new paraffin block with a 6 × 4 matrix distribution, one TMA for tumor and one TMA for healthy adjacent tissue. Two cores from renal tissue were included for orientation purposes. Then, 2 μm sections were transferred to positively activated glass slides and analyzed by immunohistochemistry.

A histological section of paraffin-embedded tumor and healthy adjacent colonic tissue of 21 patients with CRC (of 2-μm thickness) was stained with Hematoxylin/Eosin and evaluated by a pathologist, blinded to the patients's data. A score of 1 to 3 was assigned according to the tumor grade [well-differentiated (1), moderately differentiated (2) and poorly differentiated (3)], the amount of desmoplastic reaction [activation of myofibroblasts in the tumor, low (1), moderate (2), abundant (3)] and the degree of inflammatory infiltrate [low (1), moderate (2), abundant (3)]. These characteristics were considered to evaluate the association with the immunohistochemical markers and the TNM stage of patients. The presence of tumor budding (TB, or focal budding) was evaluated with the immunostaining of E-cadherin, observing foci of 5 or more tumor cells in the invasive front of the tumor, which were counted and considered positive when more than 5 foci per 20X field were observed.

### Immunohistochemistry From Histological Sections

First, sections were deparaffinized and rehydrated with deionized water. Then, they were heated in an EDTA-based buffer at pH 9.0 (Buffer EnVision Flex Antigen Retrieval, Dako, Carpinteria, CA, USA), using an electric pressure cooker for 3 min at 12–15 pounds/square inch at ~120°C, and cooled for 10 min before immunostaining. Then, all sections were incubated with 3% H_2_O_2_ (blockade of endogenous peroxidases) for 10 min and subsequently incubated with goat anti-hIL-33 (R&D Systems, Minneapolis, MN, USA), goat anti-hST2 (R&D Systems, Minneapolis, MN, USA), mouse anti-α-SMA (Sigma–Aldrich, St. Louis, MO, USA) and mouse anti-E-cadherin (BD Biosciences, Franklin Lakes, NJ, USA) primary antibody for 30 min each. Tissue sections were incubated with a goat–rabbit IgG linker (for samples incubated with goat polyclonal antibodies) and then incubated with the secondary antibody–universal polymer (EnVision Flex-HRP, Dako, Carpinteria, CA, USA). Sections were revealed with substrate + DAB and counterstained with Harris Hematoxylin. Coverslips were mounted with Tissue-Tek SCA (Sakura Finetek USA. Inc, Torrance, CA, USA). Positive and negative controls were run with each batch of patient/study slides tested.

### Immunostaining Analysis

Images were captured with Aperio ScanScope (Leica Biosystems, Wetzlar, Germany). The analysis of the images was evaluated with the Aperio ImageScope Software and the algorithm to evaluate the positive pixels was Positive Pixel Count 9. The proportion of positive pixels respective to the total pixels per area (positive and negative), were considered for association with the clinical variables and histological features [as Positivity per area, Pos/area (μm^2^)]. The Pos/area was then validated by two experienced pathologists.

### Indirect Immunofluorescence

Paraffin histological sections derived from primary CRC tumor, metastatic and healthy LNs were evaluated for the co-expression of IL-33/α-SMA and ST2/E-cadherin by immunofluorescence. Briefly, the sections were subjected to deparaffinization (NeoClear, Merck KGaA, Darnstadt, Germany), then rehydrated with a battery of alcohols from absolute ethanol to 70° ethanol. The antigenic recovery was performed with EDTA buffer (pH 8) for IL-33/α-SMA and with sodium citrate buffer (pH 6) for ST2/Ecad. Then, the sections were incubated with 100 mM glycine and 2% bovine serum albumin (BSA) (Sigma–Aldrich, St. Louis, MO, USA) plus 1% normal donkey serum in 1X PBS (Sigma–Aldrich, St. Louis, MO, USA) (for autofluorescence and non-specific proteins blocking, respectively). The sections were incubated at room temperature for 1 h with the following primary antibodies: anti-IL-33 (1/50) (Polyclonal Goat anti human, AF3625, R&D Systems, Minneapolis, MN, USA) in conjunction with anti-α-SMA (1/500) (monoclonal mouse antibody, A2547, Sigma–Aldrich, St. Louis, MO, USA) and anti-ST2 (1/100) (Polyclonal Goat anti human, AF523, R&D Systems, Minneapolis, MN, USA) in conjunction with anti-E-cadherin (1/500) (monoclonal mouse antibody, 610181, BD Biosciences, Franklin Lakes, NJ, USA). After PBS rinse, tissue sections were incubated for 1 h at room temperature with secondary antibodies (Thermo Scientific, Waltham, MA, USA) Donkey Anti-Goat IgG conjugated with Alexa Fluor 594 (1/200) and Donkey Anti-Mouse IgG conjugated with Alexa Fluor 488 (1/200). Hoechst 33342 (1/500) was used as a nuclear counterstain. Finally, slides were covered with a coverslip plus mounting solution (Dako, Agilent Technologies Inc., Santa Clara, CA, USA). Slides were visualized by C2+ confocal microscope at 20x and 60x objectives (Nikon Instruments Inc, Melville, NY, USA).

### Microarray Analysis of IL-33 mRNA in CAFs

The IL-33 mRNA expression levels from a group of colorectal CAFs by RNA microarray (GEO accession number GSE51257), were re-analyzed to correlate the IL-33 expression levels and their promigratory potential. This microarray was initially analyzed to establish a functional heterogeneity of CAFs based on their ability to induce cell migration in the CRC adenocarcinoma cell lines LIM1215 and SW480 (42).

### RNA Extraction and RT-qPCR

HT29 and HCT116 cell lines were stimulated with 50 ng/mL of rhIL-33 (R&D Systems, Minneapolis, MN, USA) for 6 h, and the expression analyses were performed using real-time qPCR (RT-qPCR). Total RNA from each sample was extracted with RNEasy Mini kit (Qiagen, Hilden, Germany) following the manufacturer's protocol, integrity was analyzed by electrophoresis in 1% agarose gel and concentration was determined by spectrophotometric analysis (Synergy 2, Biotek Instruments, Inc., Winooski, VT, USA). Then, two μg of RNA was used to synthesize cDNA using oligo-dT (Thermo Fisher Scientific, Waltham, MA, USA) and RT-affinity Script enzyme (Agilent Technologies Inc., Santa Clara, CA, USA) in a final volume of 20 μL. All mRNAs expression analyses were performed by real-time qPCR (RT-qPCR) using the Brilliant® II kit SYBR® Green QPCR Master Mix (Agilent Technologies Inc., Santa Clara, CA, USA) and primers for E-Cadherin, N-Cadherin and Vimentin at a final concentration of 250 nM in a final volume of 20 μL with 100 ng of cDNA. Amplification was performed with Mx3000 P QPCR System (Agilent Technologies Inc., Santa Clara, CA, USA). 18s was used as a reference gene to normalize mRNA levels. To analyze qPCR results 2^−ΔΔ*CT*^ method was used.

### Cell Viability Assay

HT29 and HCT116 cell lines were cultured at 2 × 10^5^ cells/well (in a 12-well plate) in DMEM medium with two different concentrations of FBS (10 and 0.5%). Cells containing each concentration of FBS were stimulated with 50 ng/mL recombinant human IL-33 (R&D Systems, Minneapolis, MN, USA). Cells were counted with trypan blue staining in a Neubauer chamber at 6, 12, 24, 48, and 72 h post-stimulus.

### Wound Healing Assay

HT29 and HCT116 cell lines stimulated with 50 ng/mL of rhIL-33 (R&D Systems, Minneapolis, MN, USA) were cultured at 6 × 10^5^ and 7 × 10^5^ cells/well, respectively (24-well plates) with DMEM 10% FBS medium, 100 U/ml penicillin, 100 μg/ml streptomycin (Gibco, Thermo Scientific, Waltham, MA, USA) until complete adherence to the plate (6 and 9 h for HT29 and HCT116, respectively). Then, medium was replaced by DMEM 0.5% FBS, 100 U/ml penicillin, 100 μg/ml streptomycin (Gibco, Thermo Scientific, Waltham, MA, USA) until the next day. Then, the wound was made with a 24-well SPL Scar Scratcher (SPL Life Sciences, Naechon-Myeon, Pocheon, South Korea). After two gentle washes with PBS to remove the detached cells, the medium was replaced with 1 mL of fresh medium with low FBS (0.5%) together with rhIL-33, except for two wells with FBS 10% or 5 ng/mL TGFβ, as a positive control. Images were acquired with Cytation 3 Cell Imaging Reader (Biotek Instruments, Inc., Winooski, VT, USA). Subsequently, the plate was incubated at 37°C/5% CO_2_ and visualized at 24 h in the same coordinates. The analysis was performed with the ImageJ software by calculating the proportional free area at 24 h normalized to time zero and comparing each stimulus with the control at the respective times.

### Statistical Analysis

First, the D'Agostino & Pearson test was used to evaluate normality of data from ELISA results. In those with normal distribution, the results were expressed as means plus standard deviation using unpaired *t*-test or one-way ANOVA for the comparison of quantitative variables. In the non-parametric data, the results were expressed in median plus interquartile range. For the comparison of paired and unpaired data between two groups, the Wilcoxon and Mann Whitney tests were used, respectively. To compare more than two groups the Kruskal Wallis test was used. The Chi square test was used for contingency analysis. The associations of the histopathological characteristics with the positivity index of the IL-33/ST2 markers in epithelium and stroma were evaluated by linear regression and Spearman coefficient. The correlation between the IL-33 transcript and migration induction was determined with a linear regression test. A *p*-value <0.05 was considered significant. A cluster analysis among Pos/area from IHC allowed us to observe those markers related to each other and grouped according to the degree of desmoplasia. The distances between the column clusters and the Heatmap rows were hierarchized using Euclidean and Ward distance methods (unsquared). Then, a principal component analysis (PCA) was applied through the Clustvis web platform, (https://biit.cs.ut.ee/clustvis/), grouping together, those tumor markers that explain the variability observed in patients with different levels of desmoplasia.

## Results

### Clinical and Histopathological Features

Table 1 summarizes clinical information from the total 62 CRC patients included in this study, divided by type of sample. Thirty patients were diagnosed with local metastasis to lymph nodes and one patient diagnosed with distant metastasis (TNM stage III and IV, respectively). Over sixty percent (66.1%) of patients had left-sided CRC.

### Tumor IL-33 From Left-Colon CRC Patients Increases in TNM Stage With Lymphatic Metastasis

**Figure 1:**
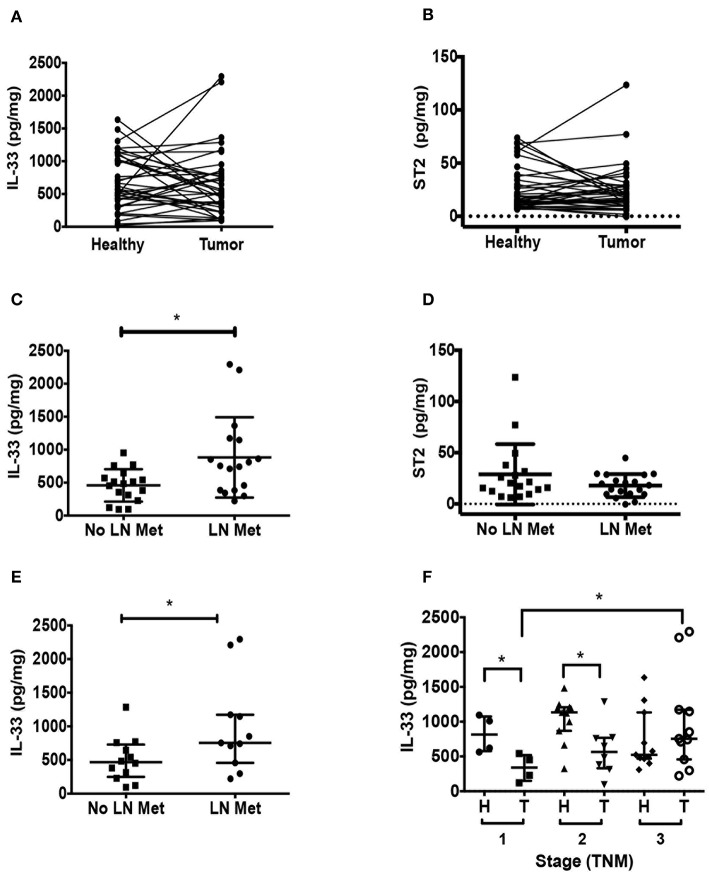
IL-33 is increased in tumors from left-colon colorectal cancer (CRC) patients with lymph node (LN) metastasis. Total IL-33 **(A)** and ST2 **(B)** protein levels were determined by ELISA in tumor and non-tumor tissue lysates from 38 CRC patients by TNM staging, Wilcoxon paired test were performed in both analyses. Total IL-33 **(C)** and ST2 **(D)** protein levels in tumor lysates from patients with and without LN metastasis using *t*-Student test to compare between groups. In **(E)**, tumor IL-33 protein levels from left-sided CRC patients with and without LN metastasis. In **(F)**, tumor and non-tumor IL-33 protein levels from left-sided CRC patients classified by TNM stage, Kruskal Wallis and Dunn's multiple comparisons post-test were performed. *P < *0.05*.

First, we evaluated IL-33 and ST2 levels in tumor, distant non-tumor tissue and plasma from CRC patients. We found similar levels between tumor IL-33 compared to distant healthy tissue (Figure 1A), as well as IL-33 levels according to TNM staging (Supplementary Figure 1A). Similar results were found with ST2 analysis (Figure 1B). Interestingly, tumor IL-33 levels were increased in those patients with LN metastasis (Figure 1C). Conversely, we found comparable ST2 levels between tumor and healthy tissue, regardless of presence of LN metastasis and TNM stage (Figure 1D and Supplementary Figure 1B, respectively).

Considering that tumor localization has been reported to impact on immune response and CRC patient outcomes (43), we analyzed separately IL-33 and ST2 levels in left-sided or right-sided CRC. In general, IL-33 levels are increased in left-sided CRC compared to right-sided CRC (Supplementary Figure 1C). Here again, we found that tumor IL-33 levels from left-sided CRC patients were increased in those with LN metastasis (Figure 1E, *p* = 0.04). At early stages, tumor IL-33 levels were lower than its corresponding non-tumor distant tissue (*p* = 0.02 and 0.03 for TNM stage 1 and 2), however, stage 3 tumor tissue show increased IL-33 compared to stage 1 (*p* = 0.03, Figure 1F). ST2 levels did not show any difference in the same analysis (Supplementary Figure 1D). Right-sided tumor IL-33 and ST2 levels were similar in those patients with regional LN metastasis compared to those without metastasis (Supplementary Figures 1E,F, respectively).

Circulating IL-33 levels evaluated from plasma were similar between CRC patients vs. controls (Mean ± SD (pg/mL): 350 ± 386.9 vs. 226.2 ± 35.41, respectively. Mann Whitney test, *p* = 0.0547), with five patients showing higher IL-33 plasma levels than the rest of patients and controls, being mostly (4/5) of pT3 stage and left-sided CRC. The other patient had right-sided CRC but with LN metastasis. Similarly, circulating ST2 levels did not show differences between patients vs. controls (Mean ± SD (pg/mL): 380 ± 303.4 vs. 391.6 ± 194.8, respectively. Mann Whitney test, *p* = 0.33). As for IL-33, ST2 plasma levels of 4 patients were detected as outliers with levels higher than controls and the rest of the patients. However, clinically, these patients were completely different from each other.

### IL-33 and ST2 Expression in Epithelial and Stromal Compartments of Primary Tumors From CRC Patients

Since we analyzed total IL-33 and ST2 protein content, we therefore evaluated IL-33 and ST2 distribution in stroma and epithelium in a tissue microarray. An initial association analysis showed that both the degree of differentiation and the amount of desmoplasia are associated to the tumor stage (TNM) (Chi-Square test, *p* = 0.0015 and 0.02, respectively), in contrast to the lymphocytic inflammatory infiltration (Supplementary Figure 2A). In addition, CRC patients with abundant desmoplasia are associated with a higher proportion of lymphatic metastasis (*p* = 0.012) (Supplementary Figure 2B).

**Figure 2:**
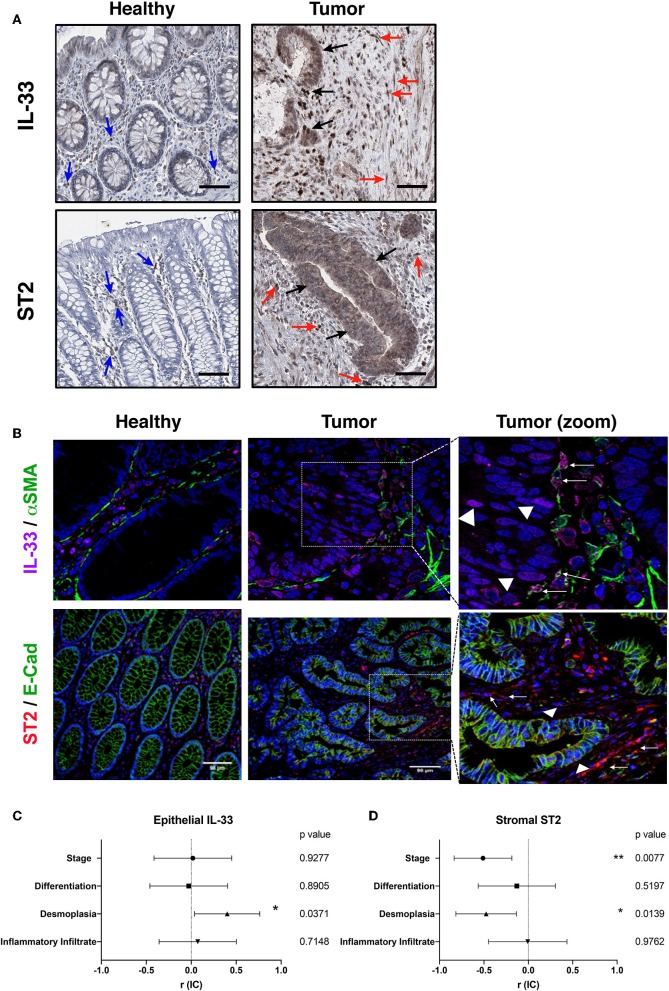
IL-33, ST2, α-SMA, and E-cadherin tissue distribution in human CRC samples. Representative images of IL-33 and ST2 **(A)** distribution in healthy colon and tumor by immunohistochemistry (in brown) (20X objective). Blue arrows indicate positive cells in healthy lamina propria, black arrows indicate IL-33^+^ or ST2^+^ tumor cells and red arrows indicate IL-33^+^ or ST2^+^ stromal cells (as may be the case). In **(B)**, co-expression by indirect immunofluorescence of IL-33 (magenta), α-SMA (green), and ST2 (red) with E-cadherin (green), Hoechst was used as nuclear counterstain (blue). Right images are a zoom from the depicted square, objective 20X. Arrows indicate fibroblast-like α-SMA^**+**^ cells IL-33^**+**^ or ST2^**+**^ (as may be the case). Triangles indicate epithelial tumor cells IL-33^**+**^ or ST2^**+**^. In **(C,D)**, Spearman correlation analyses were performed between positivity per area index of epithelial IL-33 or stromal ST2 immunostaining and histological features (TNM staging, differentiation grade, amount of desmoplasia and amount of inflammatory infiltrate), “r” coefficient and interval of confidence are depicted for each variable. *P < *0.05*, **P < *0.01*.

The content of IL-33 in healthy colon was limited to endothelial nuclear staining and some cytoplasmic staining in lamina propria mononuclear cells (LPMNCs), and scarcely observed in epithelium (Figure 2A). While the content of α-SMA in healthy colon was limited to subepithelial myofibroblasts, vessels and the muscularis mucosa (Supplementary Figure 2C), the co-expression of both markers was only observed in blood vessels (Figure 2B). In the tumor, IL-33 distribution is heterogeneous among patients, observing both nuclear and cytoplasmic staining in tumor epithelial cells, with stromal staining in fibroblast-like cells, mononuclear cells and endothelium (Figure 2A). Co-expression of IL-33 and α-SMA in tumors with advanced invasion (higher than pT3) showed a high proportion of IL-33^+^/α-SMA^+^ cells, suggesting that CAFs can express IL-33 in more invasive stages (Figure 2B).

The ST2 content in healthy colon was observed mainly in LPMNCs, being almost absent in epithelium (Figure 2A). E-cadherin distribution was only limited to healthy epithelium and homogeneous in all samples (Supplementary Figure 2C), while co-expression of both markers in healthy colon was not observed (Figure 2B). In tumor, ST2 expression was heterogeneous among patients and cytoplasmic staining of epithelial tumor cells as well as in fibroblasts and mononuclear cells in stroma (Figure 2A). The co-expression of ST2 and E-cadherin in the advanced invasion tumors (pT3) was observed mainly in some areas of epithelial tumor cells, although not necessarily co-localized in the same subcellular compartment (Figure 2B).

### Epithelial Tumor IL-33 and ST2, and Stromal ST2 Correlates With α-SMA and Desmoplasia by Hierarchical Analysis and PCA

First, the association analysis of the IL-33/ST2 distribution in tumor epithelium shows that IL-33 immunoreactivity is directly associated with a greater amount of desmoplasia (Figure 2C), observing a similar trend with ST2 immunoreactivity (Supplementary Figure 3A). Both markers are moderately correlated (Spearman *r* = 0.5, *p* = 0.01), suggesting that IL-33 and ST2 variants present in the tumor are related to fibroblast activation and could potentiate the desmoplastic reaction of the tumor. ST2 immunoreactivity in the stroma was found to be inversely associated with stage and with the amount of desmoplasia (Figure 2D).

**Figure 3:**
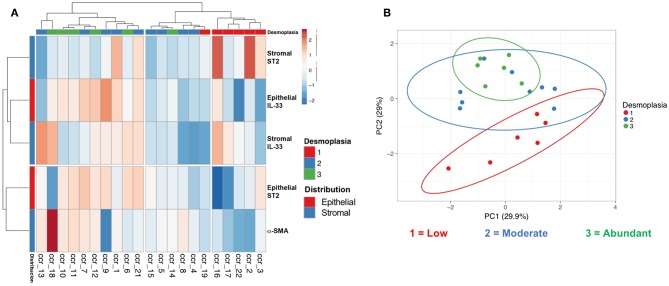
Hierarchical distribution and principal component analysis of epithelial and stromal IL-33, ST2, and α-SMA markers with desmoplasia. Clustering **(A)** and principal component analysis **(B)** including stromal and epithelial IL-33/ST2 and α-SMA immunoreactivity (with extent of desmoplasia) allowed us to distinguish clusters of low, intermediate and abundant desmoplasia.

Analysis according to the degree of desmoplasia allowed to classify three important clusters (Figure 3A), the first concentrates the cases of abundant and moderate desmoplasia, the third concentrates the cases of scarce desmoplasia; with an intermediate one that has different degrees of desmoplasia. This hierarchization shows a close relationship between variations of tumor and stromal IL-33 content together with stromal ST2 in relation to desmoplasia. Alternatively, the content of tumor ST2 shows a smaller distance or variability with the content of α-SMA. The markers together were then evaluated with a PCA, determining components (set of markers) that allow data grouping according to the variability intra and inter groups. This analysis allowed us to classify the patients who showed scarce (1) from abundant desmoplasia (3) (Figure 3B).

### Decreased Stromal ST2 and Poor Prognosis Factors Correlates With M2 Macrophage Markers in CRC Patients

**Figure 4:**
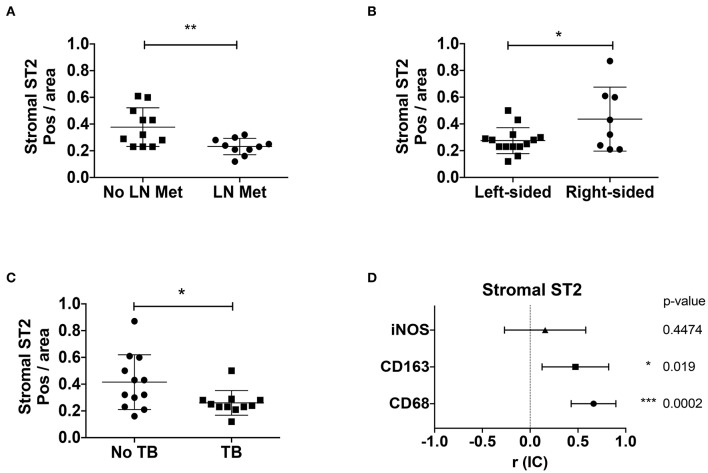
Stromal ST2 is associated to poor prognosis factors and M2 macrophages in CRC patients. Positivity per area index (AU, arbitrary units) were evaluated from stromal ST2 immunostaining in tumor samples from CRC patients by presence of LN metastasis **(A)**, localization of tumor **(B)**, tumor budding **(C)**, histological positivity per area index from stromal ST2 correlated with macrophage markers **(D)**. Mann Whitney and Spearman correlation analyses were performed. *P < 0.05, **P < 0.01, ***P < 0.001.

Since stromal ST2 showed inverse correlation with stage and amount of desmoplasia, we evaluated other clinical factors involved in CRC prognosis, such as LN metastasis, tumor budding (TB) and macrophage markers (general marker CD68, M2-marker CD163, and M1-marker iNOS), analyzed by IHC. ST2 immunoreactivity in the stroma is diminished both in patients with lymphatic metastasis [Figure 4A, Mean positivity per area ± SD (Pos/area AU, arbitrary units): 0.232 ± 0.06 vs. 0.377 ± 0.14 without LN metastasis, respectively. Student *t* test, *p* = 0.0085] and in left colon tumors [Figure 4B, Mean pos/area ± SD (AU, arbitrary units): 0.275 ± 0.09 vs. 0.463 ± 0.23 in right-sided CRC, respectively, Student *t* test, *p* = 0.0359].

Alternatively, patients with tumor budding, possibly a poor prognosis factor, were associated with abundant desmoplasia (Supplementary Figure 3B, Chi-square for trends, *p* = 0.02) and decreased stromal ST2 [Figure 4C, Mean pos/area ± SD (AU): 0.26 ± 0.091 vs. 0.415 ± 0.204 without TB, *p* = 0.031]. Interestingly, patients with TB also showed increased α-SMA (Supplementary Figure 3C, *p* = 0.009) and decreased E-cadherin (Supplementary Figure 3D, *p* = 0.001) positivity per area.

We also evaluated the positivity per area of stromal ST2 associated with macrophage markers, observing a direct association between stromal ST2 with CD68 and CD163, suggesting that a M2-macrophage rich milieu would be important in ST2 stromal expression, at least on early stages (Figure 4D and Supplementary Figure 3E).

### IL-33/ST2 Distribution in Metastatic CRC Ganglia Resembles Primary Tumor Distribution

In the healthy LN, the content of α-SMA is limited to walls of blood vessels, the content of IL-33 to the nucleus of endothelial cells (scarcely in mononuclear cells), ST2 in the cytoplasm of mononuclear cells and E-cadherin is absent (Supplementary Figure 4).

**Figure 5:**
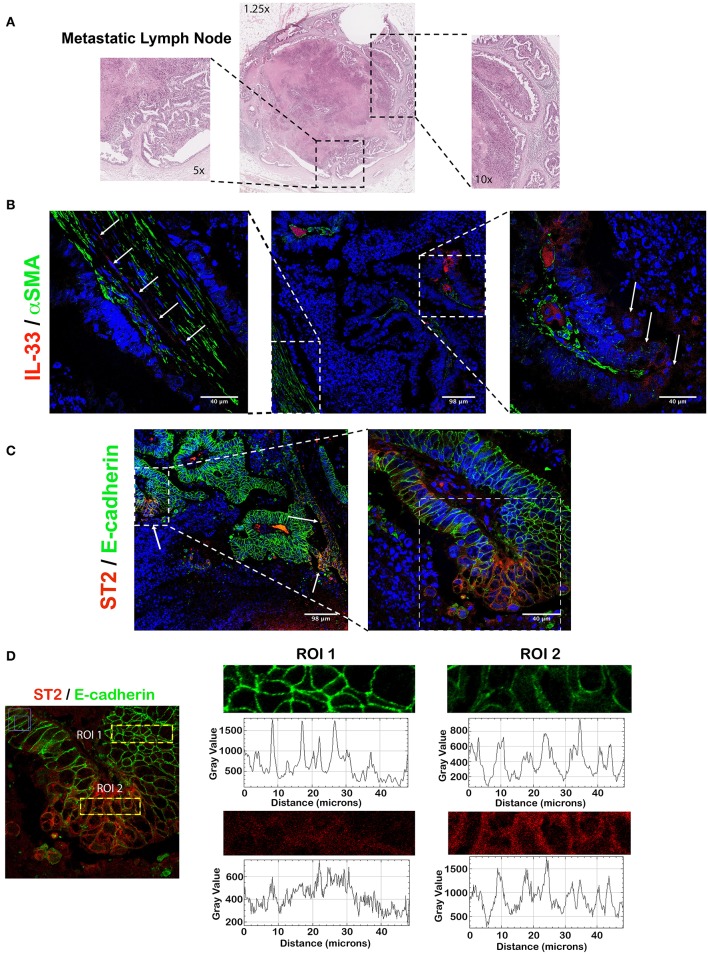
IL-33 and ST2 from metastatic LNs show similar cellular distribution than primary tumor in CRC patients. Representative image of IL-33 and ST2 localization evaluated by IFI in a CRC metastatic LN. In **(A)**, H&E staining showing high necrosis and abundant desmoplasia. In **(B)**, IL-33 (red) is expressed by both metastatic tumor cells and α-SMA^+^ cells in desmoplastic areas (green). In **(C)**, ST2 (red) is expressed in patches by metastatic tumor cells, with an intensity inversely correlated to E-cadherin membrane expression (green) **(D)**.

The metastatic LN (Figure 5A) presents variable α-SMA (desmoplasia) and IL-33 is present in cells with fibroblast-like morphology in areas with high desmoplasia and tumor invading cells (Figure 5B). Almost all of the tumor cells expressed membrane E-cadherin and in some areas the tumor cells express cytoplasmic ST2 (Figure 5C); however, localization of membrane E-cadherin decreases in the tumor cells that co-express higher intensity of ST2 (Figure 5D).

These results suggest that IL-33 and ST2 content in metastatic LN resembles to primary tumor and ST2 expression in the tumor epithelium (with reduced E-cadherin content) could be related to activation of a mesenchymal phenotype and potential tumor progression.

### IL-33 Induces a Migrating Mesenchymal Transcript Profile in HT29 Cells

**Figure 6:**
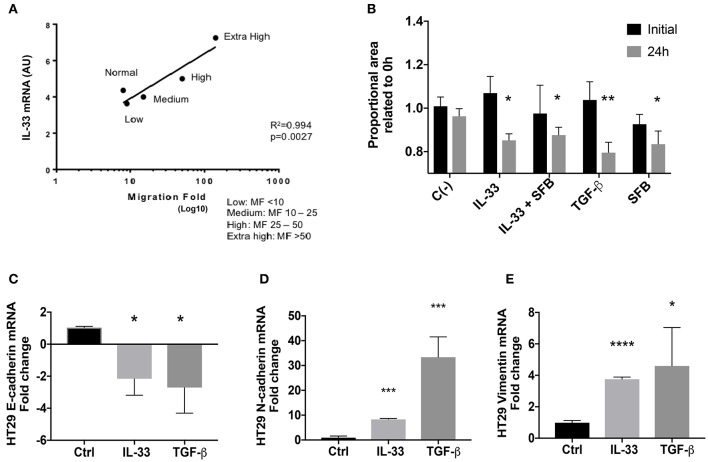
IL-33 transcript correlates with a mesenchymal phenotype in HT29 cells. In **(A)**, IL-33 transcript levels from CRC patients-derived CAFs are associated with cell migration induced by CAFs conditioned media (Lineal regression test, *R*^2^ = 0.99, *p* = 0.0027). In **(B)**, IL-33 induces HT29 cell migration at 24 h and decreases mRNA transcripts from E-cadherin **(C)** and increases N-cadherin **(D)** and vimentin **(E)** transcript levels. Student *t*- test was performed for these analyses, *P < 0.05, **P < 0.01, ***P < 0.001, ****P < 0.0001.

Initially, IL-33 transcript levels from a previous study (extracted from an analysis of mRNA microarray) correlated directly with the ability of conditioned media from colorectal CAFs to induce the migration of tumor lines of colon adenocarcinoma (Figure 6A). Then, the effect of IL-33 on the migration of the HT29 and HCT116 cell lines was evaluated through the wound closure assay at 24 h. TGFβ and FBS 10% were used as controls. At 24 h, cell proliferation was increased in high FBS but not under low FBS conditions (Supplementary Figures 5A–D). Therefore, HT29 and HCT116 cell lines were stimulated with IL-33 (50 ng/mL) with low FBS or IL-33 with 10% FBS (IL-33 + FBS), TGFβ (5 ng/mL), or 10% FBS. IL-33, in low FBS, favors the migration of HT29 cells at 24 h compared to control (Figure 6B), this effect does not occur in HCT116 cells (Supplementary Figure 6A). After, HT29 and HCT116 cell lines were exposed to IL-33 (50 ng/mL) and TGFβ (5 ng/mL) as a positive control, the transcripts of E-cadherin, N-cadherin and Vimentin were determined. IL-33 decreases the mRNA of E-cadherin in both cell lines, additionally increasing N-cadherin and vimentin mRNA only in HT29 cells, not in HCT116 cells (Figures 6C–E and Supplementary Figures 5C,D, respectively). These results suggest that IL-33 activates the onset of the mesenchymal phenotype in HT29 cells.

## Discussion

In the present study, we observed an association between tumor and stromal IL-33 and ST2 localization with desmoplasia in CRC patients, coupled with IL-33 increased in left-sided CRC patients with LN metastasis. Also, stromal ST2 was decreased in patients with left-sided cancer and LN metastasis and inversely correlated with desmoplasia, suggesting that IL-33/ST2 axis participates in CRC desmoplasia and tumor progression in this subgroup of patients.

The total content of IL-33 and ST2 were similar in tumor and normal tissue, however, it seems to depend, for IL-33, of the TNM stage. This is the first report describing quantitative analysis of tissue IL-33/ST2, since previous literature mainly supplied sera, stool, and tissue approximations by qPCR and immunohistochemistry (IHC) (34, 38, 39, 44–46). Most studies attribute a protumorigenic role to the IL-33/ST2 axis in human, murine and *in vitro* models, with increased levels of IL-33 and ST2 transcripts in tumor vs. healthy tissue from CRC patients (34, 36, 39). According to these antecedents, IL-33 increase was observed mainly in adenomas (low grade adenocarcinoma and in tumors of stages I–III), decreasing later in stage IV. In our study, we did not observe differences in IL-33 content according to the tumor stage. However, we must consider that we did not have a representative number of patients in stage IV (in part because ~20% of patients are diagnosed at this advanced stage), and of these, a low percentage is a candidate for surgery as a curative option (47). Additionally, the similarity in IL-33 protein levels between tumor and non-tumor tissue might be showing a low-grade inflammation in healthy tissue, that is undetected by histological analysis.

The immunoreactivity of IL-33 was not associated with the TNM stage or the degree of differentiation, however, recent studies show an association between IL-33 expression in the tumor epithelium of metastatic CRC and a shorter survival, suggesting that the tumor IL-33 expression is clinically important in CRC progression (37). ST2 immunoreactivity in tumor epithelium was not associated with the stage or differentiation degree, however, stromal ST2 was inversely associated with stage. Reduced ST2 might be related to the immunosuppressive milieu favored by the tumor microenvironment (48), since *in vitro* studies have shown that sST2 expression is induced by pro-inflammatory cytokines, such as IL1β and TNFα (49). Consistent with our observations, one study reported a decrease in ST2L and sST2 transcript levels in tumor with respect to adjacent normal tissue, also continuing to decrease during progression to later stages (38, 44). The discrepancies described in the papers on the role of IL-33/ST2 axis in intestinal tumorigenesis may be associated to variations in experimental design, models utilized (37, 39, 44, 45), use of chemotherapy in patients (44), as well as differences in their expression in the tumor epithelial cells, tumor microenvironment or tumor location (50). However, there are some studies that propose an antitumor role of IL-33/ST2 which might depend on the tumor microenvironment and in specific stages of tumor development and progression, as some recent comprehensive reviews have described (51–55). Therefore, protumor or antitumor role of IL-33/ST2 axis remains controversial.

In relation to IL-33/ST2 distribution, epithelial IL-33 was directly associated with a higher degree of desmoplasia while stromal ST2 was inversely associated. The presence of desmoplasia is an exacerbated reaction of myofibroblast activation (given by the content of α-SMA) in the tumor microenvironment, and it is a poor prognostic factor for CRC recurrence (17). In addition, desmoplasia exerts the modification of the extracellular matrix to favor tumor metastasis (13). The mechanism by which epithelial cells increase IL-33 expression is unclear (56), however, the effect of IL-33 on intestinal myofibroblasts was demonstrated in the CCD18Co cell line, activating the transcription of pathways related to a profibrotic response (34). Interestingly, it has been reported that IL-33 can enhance the recruitment and functions of different types of innate cells such as mast cells, Th2, regulatory T cells (Tregs), and innate lymphoid cells type 2 (ILC2s) (49, 57, 58). Of these, IL-33-mediated Treg infiltration has been described to contribute to an immunosuppressive milieu and poor prognosis not only in cancer, as seen in CRC and non-small cell lung cancer (NSCLC) models (58, 59), but also in murine models of chronic inflammatory diseases, such as colitis-induced colorectal cancer and allergic contact dermatitis-skin tumorigenesis, which in turn, might be prone to develop cancer (60). Also, ILC2s are of great interest due to their reported role in promoting fibrosis and desmoplasia after activation by IL-33 through the release of the profibrotic cytokine IL-13 (61). Thus, IL-33 could contribute to the development of desmoplasia by acting directly on myofibroblasts or indirectly through the recruitment and activation of immune cells like ILC2s.

The principal component analysis and cluster hierarchization allowed to separate patients with scarce desmoplasia from abundant desmoplasia by the pattern of epithelial and stromal IL-33, ST2, and α-SMA. This suggests that a certain distribution or pattern in the content of these molecules could be useful in discriminating the degree of desmoplasia especially in those intermediate cases, benefiting the patient's diagnosis and possibly the clinical management of this patients. However, a larger sample size of patients is necessary to validate this finding.

Another factor associated with tumor progression is the presence of tumor budding, which has been linked to a malignant phenotype on the invasive front, together with greater desmoplasia and higher risk of developing metastasis (62). Our findings show that the presence of TB correlates with tumors with greater desmoplasia, therefore, with an increase in stromal α-SMA immunoreactivity. The stromal ST2 immunoreactivity is diminished in tumors that present TB, also with decreased epithelial E-cadherin immunoreactivity. In the literature an association between TB and partial activation of EMT has been reported (63), with a decreased expression of E-cadherin or a modified membrane localization (64), probably due to alternative mechanisms activating canonical EMT transcription factors (Snail, Twist, Slug). These transcription factors have been observed at higher levels in the stroma, suggesting the participation of cells with mesenchymal phenotype (CAFs or completely dedifferentiated tumor cells) (64). In CRC, the activation of EMT and the formation of TB could be exerted by CAFs (desmoplastic reaction) by activating the IL-33/ST2 axis, as was observed in head and neck cancer cell lines (33), favoring metastasis to LNs. Additionally, other cells that respond to IL-33 could contribute to the activation of EMT during colorectal cancer progress, as is the case of Tregs, which were described by *Xiong and cols* as promoters of EMT in a context of radiation-induced pulmonary fibrosis (65). These findings highlight the capacity of IL-33 to exert its EMT inducing effects through the action of different types of cells, a characteristic that was also seen in the case of desmoplasia.

Systematic reviews show that patients with left colon tumors are different than patients with right colon tumors in terms of mutation profile, gene expression profiles and consensus molecular subtypes (41, 43, 66). In addition, patients with CRC with normal KRAS (67) may respond differently to immunological therapies according to the location of the tumor, which suggests that the immune responses and tumor evolution in the right and left colon may be different. Therefore, we were interested in evaluating the protein levels and distribution of IL-33/ST2 according to tumor location. In CRC left colon tissue in early stages, the IL-33 content is decreased vs. normal tissue but increased when lymphatic metastasis occurs (stage TNM 3). However, the IL-33 immunoreactivity in epithelium or stroma did not show differences in the location or association with any variable of progression. The ST2 immunoreactivity decreases in the stroma of left colon tumors and in those patients with lymphatic metastasis. According to this data together with antecedents that attribute an anti-tumorigenic role to ST2 in murine models (38, 44), we suggest that ST2 could have a protective role on early stages of tumor progression, particularly in left colon and specially soluble ST2, which might neutralize IL-33. However, further studies are needed where these variables are analyzed directly, mostly because the antibody used in IHC and ELISA does not distinguish between soluble and membrane ST2 variants. In right colon tumors no association between the IL-33/ST2 content with the variables evaluated was observed, unfortunately, sample size of right colon CRC patients was not enough to statistically validate. In left-colon tumors it has been described higher levels of EGFR ligands Epiregulin (EREG) and Amphiregulin (AREG). Also, activation of EGFR has been associated to increased IL-33 expression in murine models, which might explain the increased levels of IL-33 in left- vs. right-sided tumors (41, 56, 68). Alternatively, the relationship between stromal ST2 and macrophage M2 markers suggest that this cell population can be a source of ST2L, inducing early profibrotic events or activating desmoplasia. There is evidence that M2 macrophages and CAFs favor tumor progression collaboratively in various types of cancer (18, 19, 69). However, we cannot rule out other ST2-positive cells that might contribute to CRC pathogenesis, such as mast cells and ILC2 cells (70, 71).

The content of IL-33 in tumor was greater than in healthy tissue of the left colon of CRC patients with lymphatic metastasis, so we evaluated IL-33 and ST2 distribution (determined by co-staining by IFI of IL-33/α-SMA and ST2/Ecad) and found nuclear and cytoplasmic IL-33 localized in tumor epithelium (similar to the primary tumor) with cytoplasmic in fibroblastic cells (areas with desmoplasia, α-SMA^+^). Alternatively, those areas with greater ST2 immunoreactivity coincided with a decreased immunoreactivity of membrane E-cadherin in tumor cells. Given that IL-33 is localized in both tumor and fibroblastic cells (unlike the healthy LN), and additionally that IL-33 content is increased in the tumor of patients with lymphatic metastases, it is suggested that IL-33 could participate in mechanisms of tumor progression, either in an autocrine or paracrine fashion. In addition, ST2 immunoreactivity in tumor with decreased E-cadherin could reflect the induction of a mesenchymal phenotype leading to tumor progression. An association between IL-33 immunoreactivity in the tumor epithelium of patients with metastatic CRC and shorter survival has been reported, suggesting that tumor expression of IL-33 would be clinically important in its progression (37). In murine models of CRC, increased IL-33 levels in tumor cells induce greater hepatic metastasis, increase tumor size and, therefore, lower survival rate (36, 39). In breast and cervical cancer, increased expression of IL-33 has been observed in tumor cells from patients with LN metastases (72, 73), suggesting that IL-33/ST2 might participate in invasion and metastasis by remodeling primary and metastatic tumor microenvironment.

The effect of IL-33 on stromal activation was observed in a subepithelial fibroblast cell line (34), increasing the transcription of pathways mainly associated with TGF-β and extracellular matrix remodeling. In addition, the activation of fibroblasts according to the degree of invasion is particularly important in tumor progression, as it modifies the organization and composition of the extracellular matrix, forming pathways facilitating the migration and invasion of tumor cells (13). In patients with CRC, abundant desmoplasia is a predisposing factor to a shorter survival (74) and is also a risk factor for LN metastases (62). This suggests that one of the direct mechanisms by which IL-33 could be favoring tumor progression in CRC is through desmoplasia activation. But also, IL-33 from stromal fibroblasts can promote macrophage polarization to an M2 profile, secretion of MMP-9 and metastasis, as seen in an pancreatic adenocarcinoma model (75), and indirectly, IL-33 can also induce chemokine secretion by the tumor, recruiting M2 type macrophages (38) and contributing to greater desmoplasia.

As IL-33 content increases in patients with lymphatic metastasis, we evaluated the effect of IL-33 on the migratory capacities of the CRC cell line HT29 using the wound closure test as an approximation of the migration phenomenon (44). IL-33 induced greater migration observed at 24 h with respect to the control 0.5% FBS. There were no evaluations after 24 h since cell proliferation interferes in the measurement, while IL-33 stimulation (in the presence of FBS 10%) also increases proliferation at 24 h, again interfering with the wound test. Additionally, and since ST2 immunoreactivity coincides with a decrease of E-cadherin in metastatic lymphoid tumor cell membrane, we evaluated the effect of IL-33 on the transcript levels of classical EMT markers (E-cadherin, N-cadherin and vimentin). We have determined that IL-33 decreases E-cadherin transcript and increases vimentin and N-cadherin transcripts, which indicate the induction of a mesenchymal phenotype in HT29 cells. These results, coupled with the wound closure assay, suggest that IL-33 could induce a change toward a mesenchymal phenotype in HT29 cells through the activation of EMT as was described in a head and neck cancer model (33); nevertheless, the analysis of additional EMT markers is needed to confirm the involvement of this process. The effect of IL-33 was also studied on HCT116 cells (Supplementary Figures 6B–D), where we observed a downregulation of E-cadherin and increase of vimentin transcript levels in response to IL-33, but no changes in migration or in N-cadherin transcript levels, which may be due to ST2L decrease, therefore truncating the process of mesenchymal activation. Alternatively, phenotypic and molecular differences of HT29 and HCT116 lines suggest the HCT116 line needs an additional stimulus to complete the mesenchymal phenotype of EMT.

**Figure 7:**
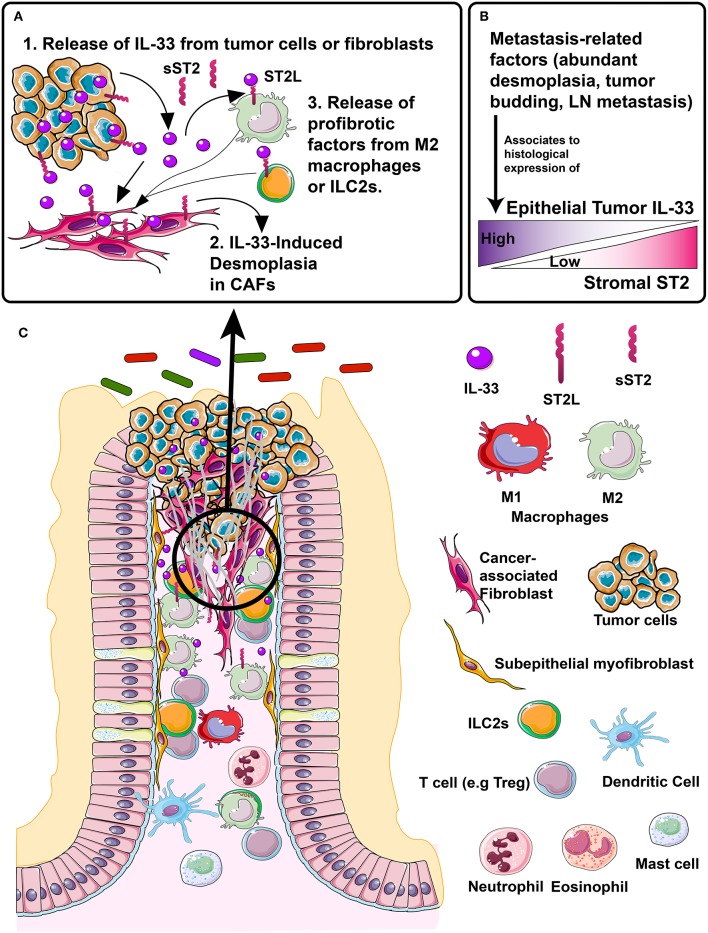
Proposed model of IL-33/ST2 axis association to desmoplasia, a metastasis-related process in colorectal cancer patients. **(A)** In left-colon tumors, different events, e.g., necrosis, inflammation, mechanical strain, can induce the release of IL-33 in the tumor microenvironment, from both the tumor epithelium and fibroblasts, (A.1). IL-33 binds to ST2 receptor located in fibroblasts, directly inducing desmoplasia (A.2). The M2 macrophage or ILC2s located in the stroma can express both sST2 (which in early stages may neutralize IL-33 effects) and membrane bound ST2L which may activate desmoplasia indirectly by secreting profibrotic factors, such as IL-13 and TGFβ (A.3). **(B)** Metastasis-related factors such as abundant desmoplasia, tumor budding and LN metastasis are histologically associated with increased/high tumor cell IL-33 expression and decreased/low stromal ST2 expression, which if validated, could become a histological signature of tumor progression. **(C)** Representation of CRC tumor microenvironment (left) with distinctive molecules and cells (right) signaling through IL-33/ST2 axis and involved in tumor progression and metastasis.

Therefore, the IL-33/ST2 axis may participate in the interaction of the tumor microenvironment, mediating processes associated with metastasis in CRC primarily in the left colon (Figure 7).

## Conclusion

In conclusion, tumor IL-33 increase and stromal ST2 decrease are associated with greater desmoplasia in left colon tumors, which in turn might contribute to the development of lymphatic metastasis. The inflammatory content of the microenvironment increases IL-33 transcript in CAFs, whose levels are associated with increased cell migration, whilst activating the onset of a migrating mesenchymal phenotype in an adenocarcinoma cell line. Controlling IL-33/ST2 axis expression represents a potential target to improve a personalized left-sided CRC therapy.

## Ethics Statement

This study was carried out in accordance with the recommendations of the Review Boards and Local Ethical Committees from Universidad de Chile Clinical Hospital, Tissue Biobank of Universidad de Chile and Clinica Las Condes, with written informed consent from all subjects. All subjects gave written informed consent in accordance with the Declaration of Helsinki and were identified only by codification established by their respective Center to keep anonymity. The protocol was approved by the Local Ethical Committees from Universidad de Chile Clinical Hospital, Tissue Biobank of Universidad de Chile and Clinica Las Condes.

## Author Contributions

GL designed and performed most of the experiments, analyses of results and manuscript drafting. MD, KD-C, DP-V, and DD-J contributed to design, discussion of results and performed IL-33 detection in human samples. MD also contributed with macrophages IHQ analyses. DR and SS performed the immunohistochemistry staining of TMA, JR validated Aperio algorithms and performed histological characterization of TMAs. CS contributed in statistical analyses. CP contributed with CAF microarray data. RQ, FL-K, UK, MA, and DS enrolled CRC patients. IG selected colorectal tissue from Tissue Biobank. M-JG and OO-S participated in the analysis and discussion of results. HC contributed with qPCR equipment and EMT expertise. GD-A and MH contributed to study design and supervised work. All the authors contributed to drafting and discussion of the manuscript.

### Conflict of Interest Statement

The authors declare that the research was conducted in the absence of any commercial or financial relationships that could be construed as a potential conflict of interest.
