# Corrigendum: Interleukin 33/ST2 Axis Components Are Associated to Desmoplasia, a Metastasis-Related Factor in Colorectal Cancer

**DOI:** 10.3389/fimmu.2019.02149

**Published:** 2019-09-24

**Authors:** Glauben Landskron, Marjorie De la Fuente López, Karen Dubois-Camacho, David Díaz-Jiménez, Octavio Orellana-Serradell, Diego Romero, Santiago A. Sepúlveda, Christian Salazar, Daniela Parada-Venegas, Rodrigo Quera, Daniela Simian, María-Julieta González, Francisco López-Köstner, Udo Kronberg, Mario Abedrapo, Iván Gallegos, Héctor R. Contreras, Cristina Peña, Guillermo Díaz-Araya, Juan Carlos Roa, Marcela A. Hermoso

**Affiliations:** ^1^Immunology Program, Innate Immunity Laboratory, Faculty of Medicine, Biomedical Sciences Institute, Universidad de Chile, Santiago, Chile; ^2^Research Sub-direction, Academic Direction, Clinica Las Condes, Santiago, Chile; ^3^Pathology Department, Faculty of Medicine, Pontificia Universidad Catolica de Chile, Santiago, Chile; ^4^Inflammatory Bowel Disease Program, Gastroenterology Department, Clinica Las Condes, Santiago, Chile; ^5^Cell and Molecular Biology Program, Faculty of Medicine, Institute of Biomedical Sciences, Universidad de Chile, Santiago, Chile; ^6^Coloproctology Department, Clinica Las Condes, Santiago, Chile; ^7^Coloproctology Surgery Department, Hospital Clinico Universidad de Chile, Santiago, Chile; ^8^Pathology Department, Hospital Clinico Universidad de Chile, Santiago, Chile; ^9^Department of Basic and Clinic Oncology, Faculty of Medicine, Universidad de Chile, Santiago, Chile; ^10^Medical Oncology Department, Ramon y Cajal University Hospital, IRYCIS, CIBERONC, Madrid, Spain; ^11^Molecular Pharmacology Laboratory, Faculty of Chemical Pharmaceutical Sciences, Universidad de Chile, Santiago, Chile

**Keywords:** colorectal cancer, cancer associated fibroblasts, interleukin 33, desmoplasia, epithelial-mesenchymal transition

“Francisco López-Köstner” was not included as an author in the published article. The corrected Author Contributions Statement appears below.

“GL designed and performed most of the experiments, analyses of results and manuscript drafting. MD, KD-C, DP-V, and DD-J contributed to design, discussion of results and performed IL-33 detection in human samples. MD also contributed with macrophages IHQ analyses. DR and SS performed the immunohistochemistry staining of TMA, JR validated Aperio algorithms and performed histological characterization of TMAs. CS contributed in statistical analyses. CP contributed with CAF microarray data. RQ, FL-K, UK, MA, and DS enrolled CRC patients. IG selected colorectal tissue from Tissue Biobank. M-JG and OO-S participated in the analysis and discussion of results. HC contributed with qPCR equipment and EMT expertise. GD-A and MH contributed to study design and supervised work. All the authors contributed to drafting and discussion of the manuscript.”

“There was also an error regarding the affiliations for Mario Abedrapo. As well as having affiliation 7, they should also have affiliation 6.”

The authors apologize for these errors and state that this does not change the scientific conclusions of the article in any way. The original article has been updated.

