# Supplementary material for: Interleukin 33/ST2 Axis Components Are Associated to Desmoplasia, a Metastasis-Related Factor in Colorectal Cancer
Source: Front Immunol. 2019 Jun 21;10:1394. doi: 10.3389/fimmu.2019.01394 (PMC6598075; doi:10.3389/fimmu.2019.01394)
Supplement: Supplementary file 1 [file Data_Sheet_1.PDF]

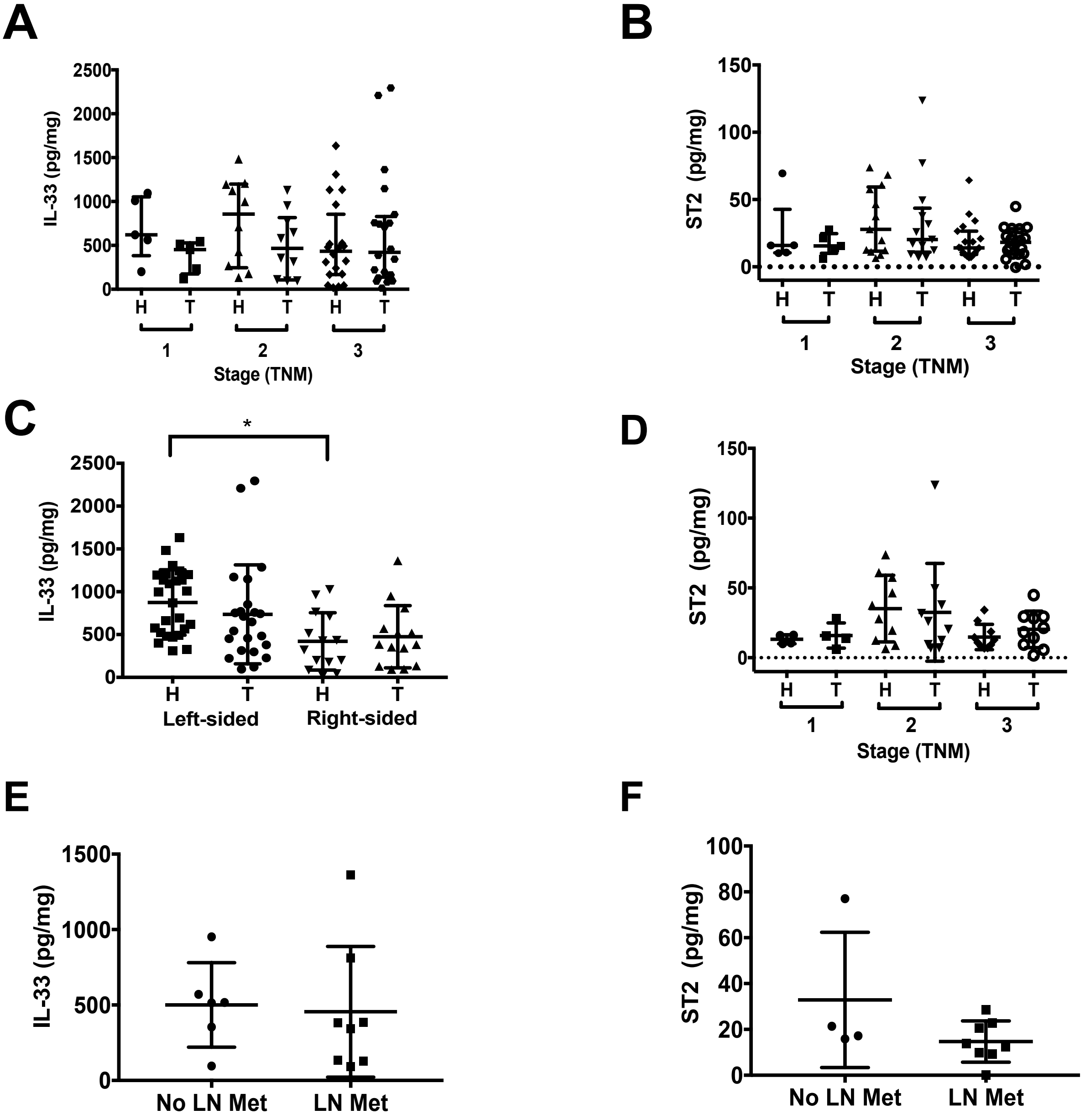

Supplementary Figure 1. IL-33 and ST2 protein levels in colorectal cancer patients

A. Total IL-33 protein levels from healthy (H) and tumor (T) mucosal lysates by TNM stage.
B. Total ST2 protein levels from healthy (H) and tumor (T) mucosal lysates classified by TNM stage.
C. IL-33 protein levels from healthy and tumor mucosal lysates in patients with left-sided CRC vs right-sided CRC.
D. ST2 total protein levels from healthy (H) and tumor (T) mucosa from left-sided colorectal cancer by TNM stage.
E. IL-33 protein levels from right-sided tumor mucosal lysates in patients without LN metastasis (No LN Met) and with LN metastasis (LN Met).
F. ST2 protein levels from right-sided tumor mucosal lysates in patients without LN metastasis (No LN Met) and with LN metastasis (LN Met).
All data are represented as Mean $\pm$ SD in pg/mg (normalized to mg total protein).

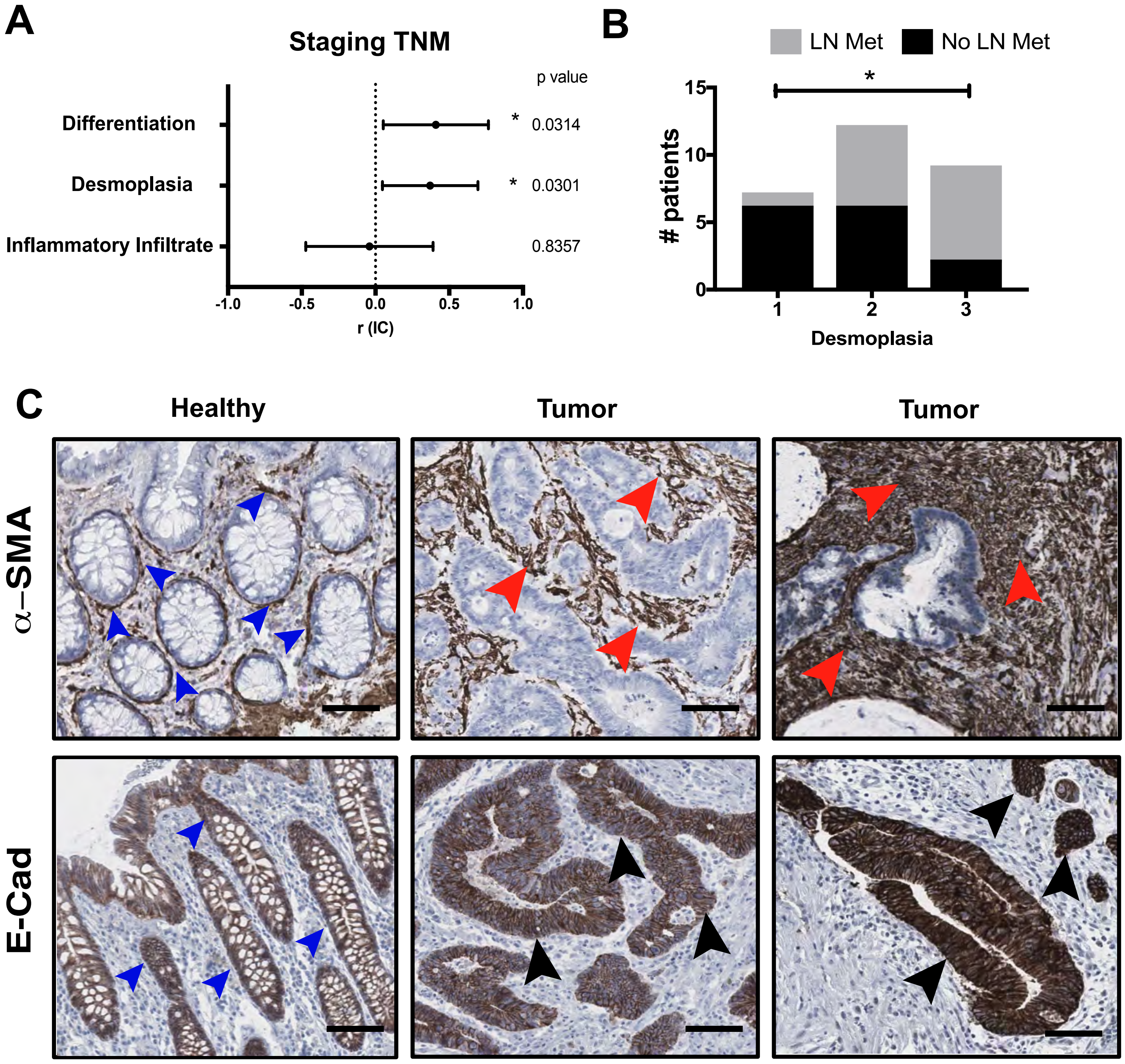

Supplementary Figure 2. Histological features from colorectal cancer patients

A. Association analysis between staging (TNM) and tumor grade of differentiation, amount of desmoplasia and amount of inflammatory infiltrate. (Spearman analysis was performed, data is expressed as r (95% interval of confidence), *$p<0.05$).

B. Frequency analysis of patients grouped by desmoplasia and classified by presence of lymph node metastasis (contingency analysis by Chi-square, *$p<0.05$).

C. Representative image of immunohistochemistry of α-SMA and E-cadherin (in brown) from healthy and tumor samples from two colorectal cancer patients (Objective 20X). Blue arrowheads indicate positive cells in healthy lamina propria, black arrowsheads indicate E-cadherin positive tumor cells and red arrowheadsindicate α–SMA positive stromal cells (desmoplasia).

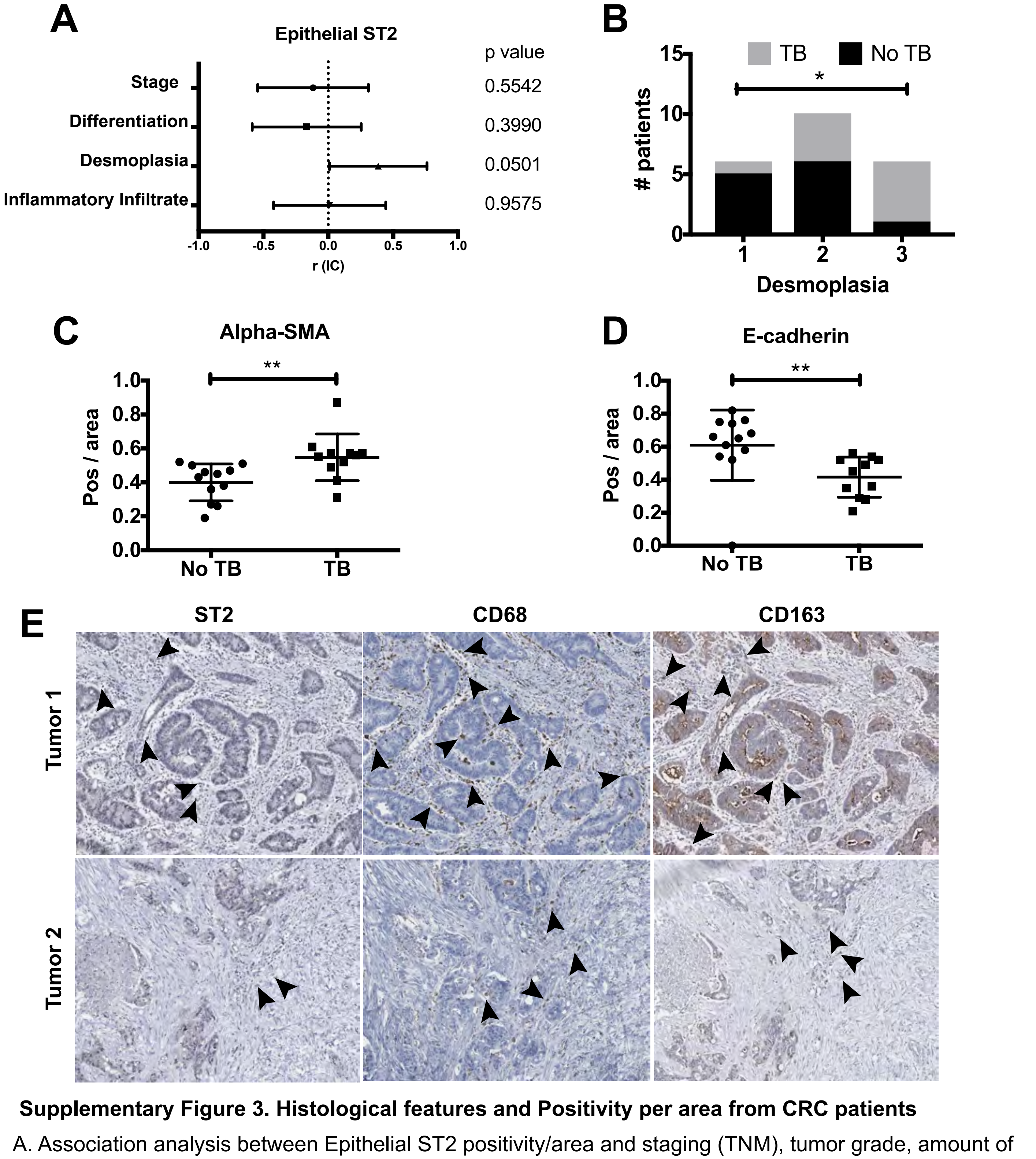

Supplementary Figure 3. Histological features and Positivity per area from CRC patients

A. Association analysis between Epithelial ST2 positivity/area and staging (TNM), tumor grade, amount of desmoplasia and amount of inflammatory infiltrate (Spearman analysis was performed).
B. Frequency of patients grouped by amount of desmoplasia and classified by presence of tumor budding (contingency analysis by Chi-square, *p<0.05).
C and D. Positivity per area of α-SMA and E-cadherin, respectively, in tumor mucosa in patients with tumor budding compared to patients without tumor budding. Mann Whitney analysis was performed, **p<0.01.
E. Representative image of ST2, CD68 and CD163 immunohistochemistry of tumor mucosa from two CRC patients. Arrowheads indicate positive cells for each marker (in brown) (20X objective).

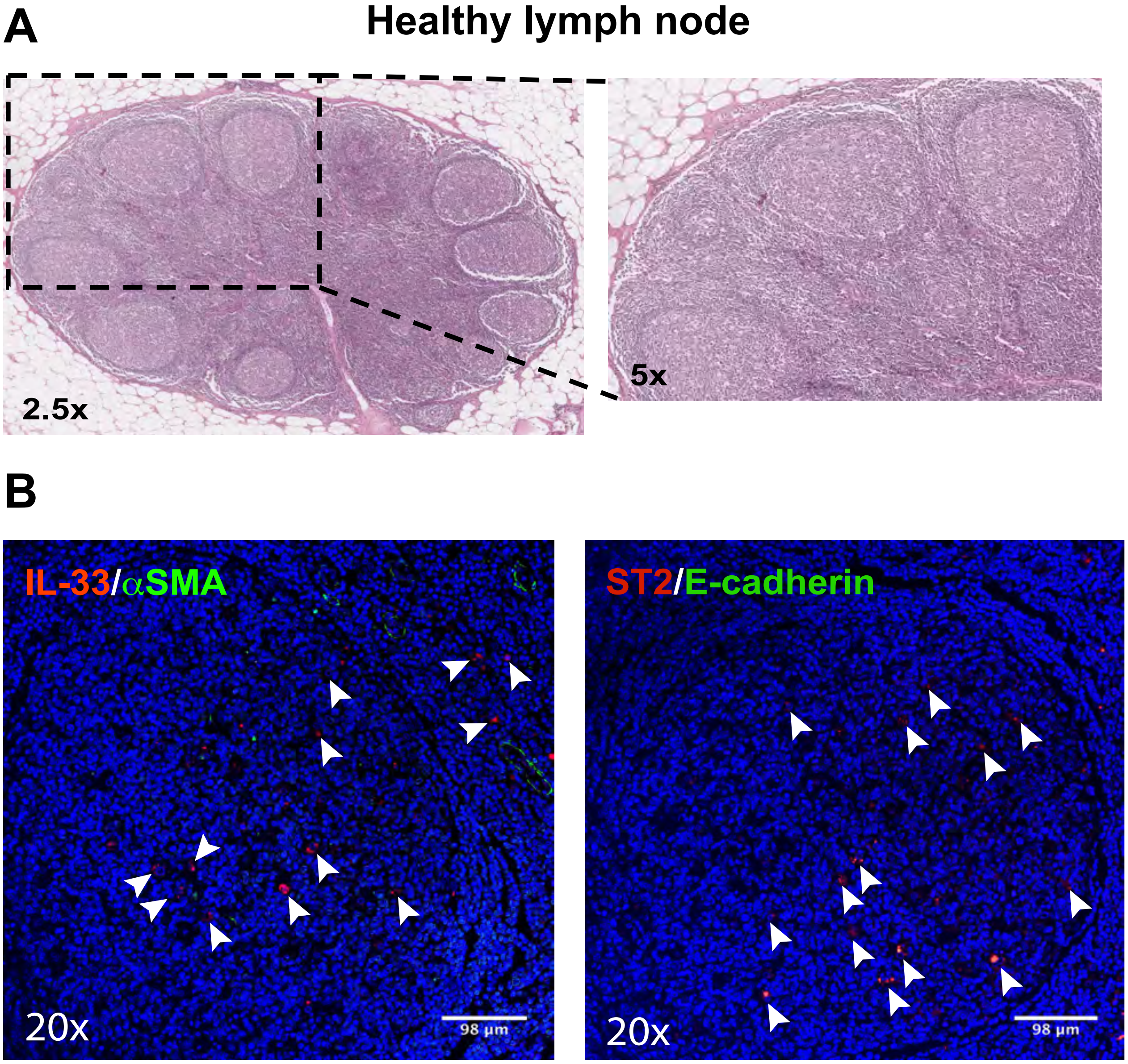

Supplementary Figure 4. Histology and IL-33/ST2 staining from a healthy lymph node
A. Representative image from Hematoxylin/Eosin staining in a healthy lymph node from a colorectal cancer patient (2.5X objective). In zoom, follicule normal structure and intact capsule (5X objective).
B. Representative image from IL-33/αSMA and ST2/E-cadherin double staining in a healthy lymph node from a colorectal cancer patient (20X objective).
Arrowheads indicate IL-33 or ST2 positive cells. There is α–SMA staining in some vascular smooth cells and absence of E-cadherin positive cells.

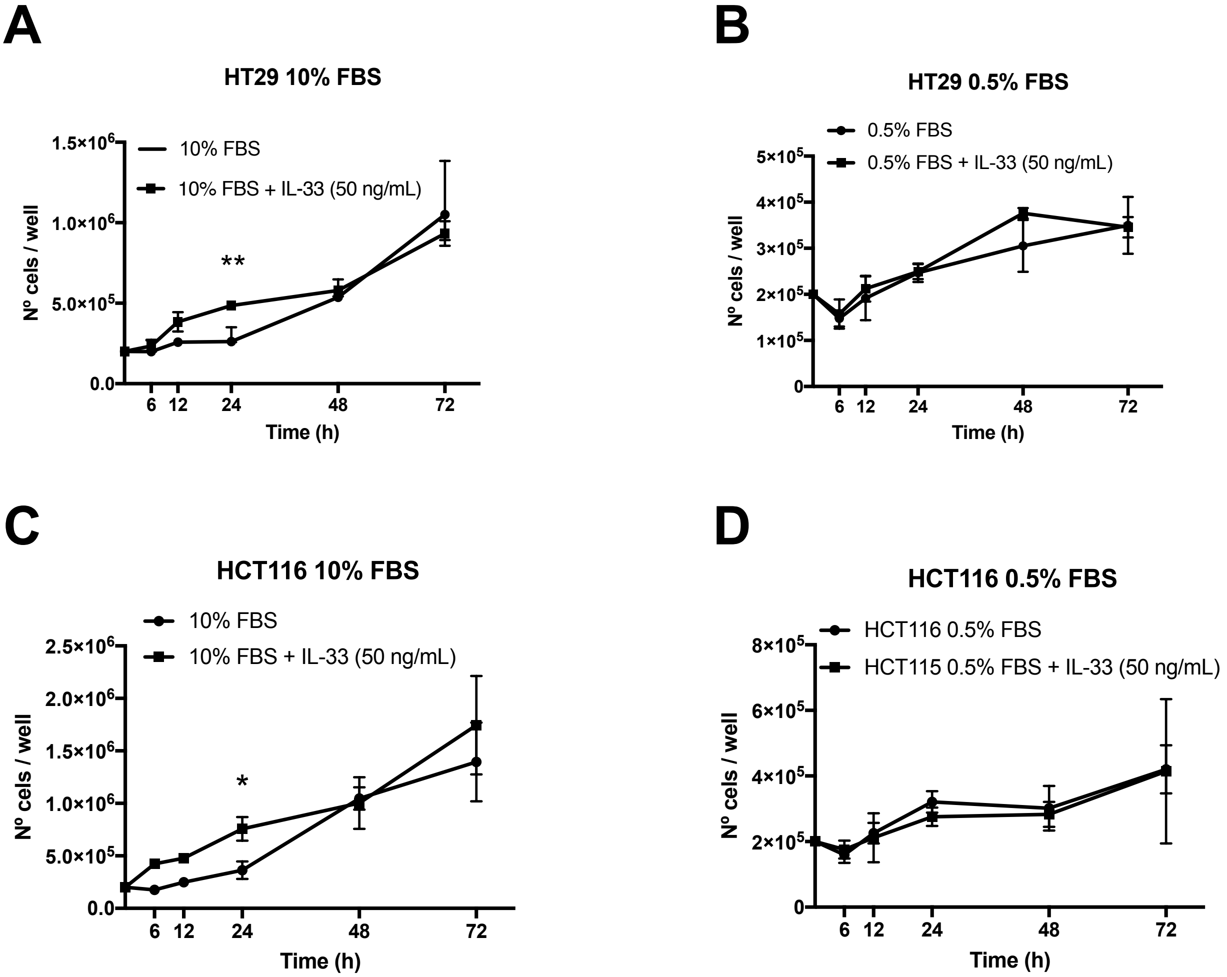

Supplementary Figure 5. Cell viability of HT29 and HCT116 cells with low FBS and IL-33

HT29 and HCT116 cells were cultured in 10% or 0.5% FBS at 6, 12, 24, 48, 72 hours. The number of viable cells were counted using trypan blue stain. Each cell count was compared with their respective control using Paired t-test, n=3. *$p<0.05$. **$p<0.01$.

A. HT29 cells stimulated with 50 ng/mL of rhIL-33 in 10% FBS.
B. HT29 cells stimulated with 50 ng/mL of rhIL-33 in 0.5% FBS.
C. HCT116 cells stimulated with 50 ng/mL of rhIL-33 in 10% FBS.
D. HCT116 cells stimulated with 50 ng/mL of rhIL-33 in 0.5% FBS.

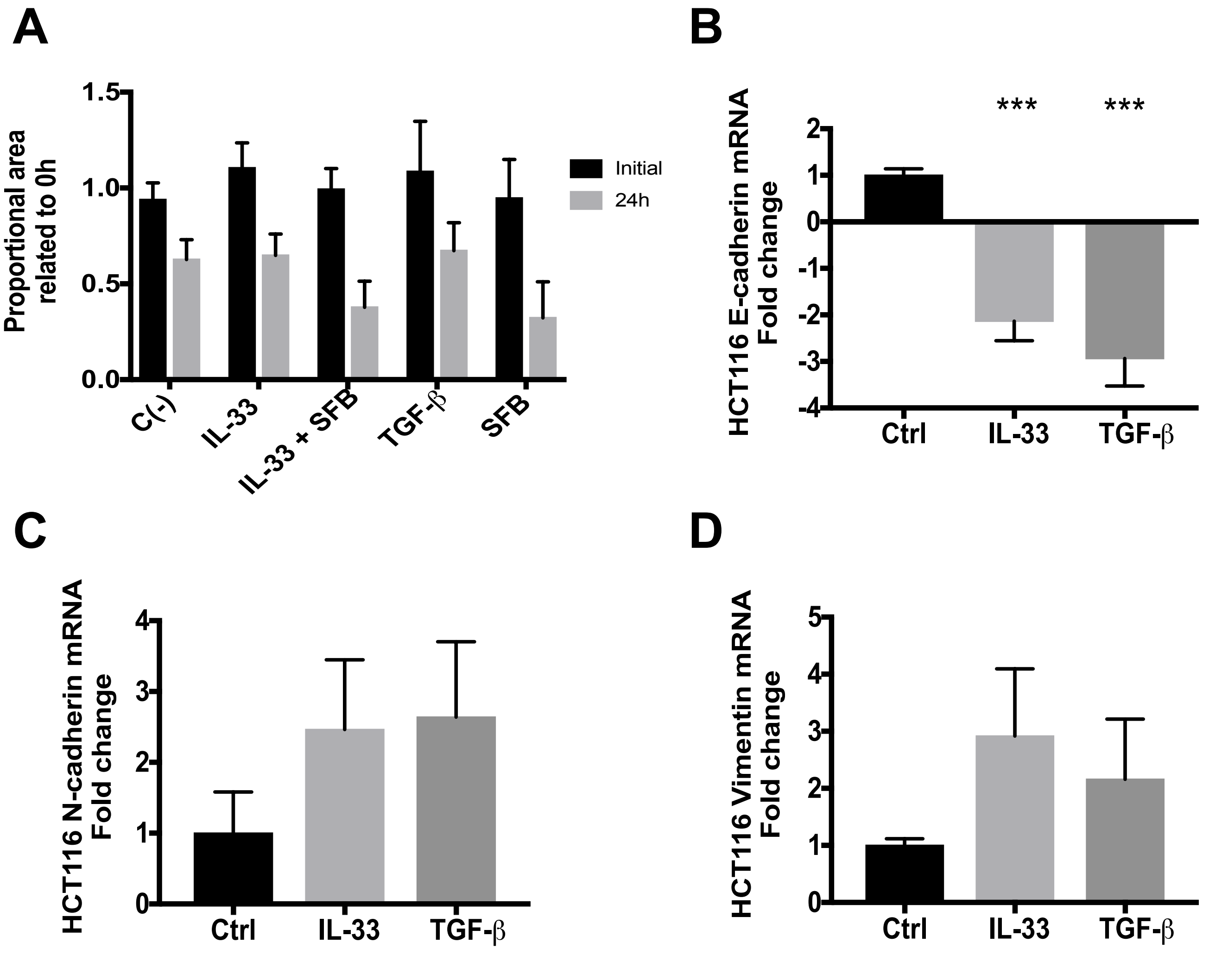

Supplementary Figure 6. Wound healing assay and epithelial mesenchymal transition in HCT116 cells

A. Wound healing assay in HCT116 stimulated with 50 ng/mL rhIL-33 or 5 ng/mL TGF-β. Proportional area of closure is depicted respect to initial time, Student t-test was performed, n=4.
B. E-cadherin transcript levels from HCT116 stimulated with 50 ng/mL rhIL-33 or 5 ng/mL TGF-β by RT-qPCR (Student t-test was performed, ***p<0.001). n=3
C. N-cadherin transcript levels from HCT116 stimulated with 50 ng/mL rhIL-33 or 5 ng/mL TGF-β by RT-qPCR (Student t-test was performed). n=3
D. Vimentin transcript levels from HCT116 stimulated with 50 ng/mL rhIL-33 or 5 ng/mL TGF-β by RT-qPCR (Student t-test was performed). n=3
